# Knowledge, Attitude and Practice towards Antibiotic Use among the Public in Kuwait

**DOI:** 10.1371/journal.pone.0117910

**Published:** 2015-02-12

**Authors:** Abdelmoneim Ismail Awad, Esraa Abdulwahid Aboud

**Affiliations:** 1 Department of Pharmacy Practice, Faculty of Pharmacy, Kuwait University, Kuwait City, Kuwait; 2 Ministry of Health, Kuwait City, Kuwait; NERC Centre for Ecology & Hydrology, UNITED KINGDOM

## Abstract

**Background:**

The emergence and spread of bacterial resistance to antibiotics is a growing problem worldwide, which presents a significant threat to public health globally in the 21^st^ century. A substantial evidence has shown that the general community plays a role in the increase and spread of antibiotic resistance. The present study was designed to determine knowledge, attitude and practice towards antibiotic use.

**Methods:**

A cross-sectional survey was performed using a pretested self-administered questionnaire on a sample of 770 randomly selected Kuwaiti individuals. Descriptive and multivariate logistic regression analysis were used in data analysis.

**Results:**

The response rate was 88.3%. Nearly three-quarters (72.8%) of respondents had been prescribed antibiotics within 12 months prior to the study period, and 36% of them had not finished the course of treatment. Over one-quarter (27.5%) were self-medicated with antibiotics to treat mainly common cold, sore throat and cough. Self-medication was more prevalent among those who were prescribed antibiotics and those who had attitudes towards using and accessing antibiotic inappropriately. Almost 47% of participants had low knowledge regarding action, use, safety and resistance of antibiotics. Forty one percent of respondents had attitudes towards using and accessing antibiotic inappropriately. Better knowledge was found to be a predictor for positive attitude. Respondents level of agreement that doctors often prescribe antibiotics to meet the patient’s expectation, and that doctors often take time to consider carefully the need for an antibiotic were 52.7% and 35.3%, respectively.

**Conclusions:**

These findings will aid in the assessment of the adequacy of present public educational campaigns. Also, it will provide further insight in designing future multifaceted interventions to promote specific messages to rationalize antibiotic use, and compensate for knowledge and attitude gaps as an effort towards preventing development of antibiotic resistance.

## Introduction

In recent decades, the emergence and spread of bacterial resistance to antibiotics is a growing problem worldwide, which presents a significant threat to public health globally in the 21^st^ century [1]. The increase of antibiotics resistance will endanger their therapeutic effectiveness, increase treatment failures and, as a result, lead to longer and more severe illness episodes with higher costs and mortality rates [2]. The inappropriate and excessive use of antibiotics are among the key factors for the increase and spread of resistance [3–5]. The improper use of antibiotics may arise from a complex interaction between numerous factors, such as prescribers’ knowledge and experiences, diagnostic uncertainty, perceptions of patients in relation to the patient-prescriber interaction, and insufficient patient education by physicians. In addition, other factors include patients’ knowledge, beliefs and attitudes towards antibiotic use, self-medication, patients’ expectations, and patients’ experience with antibiotics [6–8]. Thus, the control of antibiotic utilization needs multifaceted interventions involving knowledgeable and engaged healthcare practitioners and the public.

In 2006, a study was conducted to identify the patterns of antibiotic resistance in China, Kuwait and the USA. It revealed that Kuwait had the second rapid growth rate of resistance during the period from 1999 to 2003 [9]. Another two studies reported the emergence of antibiotics resistance in the Gulf Cooperation Council (GCC) countries including Kuwait [10,11]. This rise in the antibiotic resistance was attributed mainly to the inappropriate prescribing of antibiotics and overuse of antibiotics including self-medication. Other factors included the lack of policies for restricting and auditing antibiotic prescriptions in many GCC countries, misuse of antibiotics in the animal health sector recognizing most classes of antibiotics are used both in animal and human health, and lack of guidelines for the use of antibiotics in the animal industry [10,11]. All of these factors underscore the need for investigating and tackling such practices. In 2009, a study was conducted to investigate the prescribing practices at 50 primary healthcare centers in Kuwait. It revealed a high prescribing rate for antibiotics with almost four-in-ten prescriptions involving an antibiotic [12]. Kuwait National Campaign for the Proper Use of Antibiotics was launched in 2009 targeting physicians, pharmacists, patients and the public in government hospitals to increase knowledge about the prudent use of antibiotics. In addition, a one-day community awareness campaign was held in the largest shopping mall in Kuwait, which was covered by the media [13]. However, surveys were not undertaken before the campaign to understand the public knowledge, attitudes, and behaviors towards antibiotic use, and after the campaign to determine its impact.

A substantial global evidence has shown that the general community plays a role in the increase and spread of antibiotic resistance [5,6,14–16]. The World Health Organization (WHO) identified three key issues for public involvement: improving access to medical facilities, decreasing unnecessary use of antimicrobials, taking a full course of treatment, and not giving out medication to other people or keeping left-over medication for future needs. The WHO also urged member countries to initiate educational interventions for patients and the general population aimed at rationalizing the use of antibiotics to combat resistance [2]. Thus, improving the public knowledge and changing their attitudes towards antibiotic use will be a crucial early strategy to maintain antibiotic effectiveness. However, there are wide variations in antibiotic utilization globally, and tailoring of educational interventions necessitates understanding of the public knowledge, attitudes, and behaviors towards antibiotic use in each country. Although a number of studies have determined the antibiotic use, knowledge, attitudes, and behaviors of the general population worldwide, [5,15–26] to our knowledge there are no population studies from the GCC countries including Kuwait. Thus, this study was designed to determine knowledge, attitude and practice towards antibiotic use among the public in Kuwait. It also explored the public views on the doctors’ habits and the patient-doctor relationship when prescribing antibiotics.

## Materials and Methods

### Study design and population

A quantitative, cross-sectional study was conducted in Kuwait, a Middle-Eastern country with an area of 17,820 km^2^ and an estimated population of 3,065,850 people; 35.6% of whom are Kuwaitis (2011 estimate) [27]. The survey was conducted during the period from January to March 2014. The study population consisted of Kuwaiti individuals from six governorates of Kuwait: Al-Ahmadi, Al-Farwaniyah, Al-Jahra, Capital, Hawalli and Mubarak Al-Kabeer. The study was conducted in accordance with the Declaration of Helsinki and national and institutional standards. Ethical approval for this study was obtained from the “Human Ethical Committeee, Ministry of Health, Kuwait”.

The sample size was based on the assumption that the proportions of response to most of the main questions is 50%, as both responses and response rates were completely unknown due to the fact there are no previous similar studies from Kuwait or other GCC countries. It was determined using the Raosoft sample size calculator using a margin of error of 5%, a confidence interval of 95%, a population size of 565,303 people aged ≥ 20 years old, and an expected response of 50% [28]. The minimum sample size estimated for the study was 384. Assuming a response rate of 50%, a larger sample size of 770 Kuwaitis were enrolled in the study. The study population were selected from a number of different venues in Kuwait in order to increase the generalizability of the findings. Kuwaiti nationals from ministries, universities, schools as well as families, relatives, friends and neighbors of the Health Sciences Centre students were approached to participate in the study. In the first stage of selection, a random sampling was used to select five ministries out of ten Government Ministries in Kuwait as well as three universities out of seven in the country. Likewise, three governorates out of six were randomly selected and from each of these governorates five schools were selected to make a total of 15 schools. All Kuwaiti workers in these selected ministers, universities, and schools available on the days of data collections were invited to participate in the study. Additionally, in order increase the external validity, we also sampled subjects who were family members, relatives, friends, and neighbors of 30 students from the Health Science Center of Kuwait. These students were selected using a stratified random sample, where ten students were randomly picked from each of the three faculties. These individuals were contacted and given an explanation with regard to the purpose of the study. They were free to refuse participation in the study. Data were collected anonymously via self-administered questionnaire. Those who agreed to take part in the study were given the questionnaires, which were completed anonymously and collected afterwards. They were assured for confidentiality and gave written consent to participate in the study. Incentives were not offered for completion of the questionnaire. Exclusion criteria were expatriates, age < 21 years and ≥ 80 years.

### Study questionnaire

A literature review of similar previous studies was conducted to identify potential items for the study instrument. Based on the literature search, the study questionnaire was adapted from validated surveys that were previously used in Sweden and the United Kingdom (UK), and tailored to suit the local population and assure its applicability [15–17]. The face and content validity of the adapted questionnaire were established by a research group at Kuwait University. The questionnaire was translated into Arabic and subjected to a process of forward and backward translation. The accuracy and meaning of the translated versions both forward and backward were checked, and recommended amendments where necessary were discussed before being finalized. It was pretested for content, design, readability, and comprehension on 20 Kuwaiti individuals, and modifications were made as necessary so that the questionnaire was simple to understand and answer, yet gave accurate data.

The pre-tested questionnaire comprised of five sections, and contained both open-ended and close-ended questions. The first section recorded the respondents’ socio-demographic characteristics such as age, gender, marital status, educational level, employment, and residence (Table 1). Section two consisted of nine close-ended questions to provide information about the practice of antibiotic use. It included the following questions: whether they had been prescribed an antibiotic within 12 months prior to the study period, frequency of antibiotic prescribing during the last year, whether they had finished their last antibiotic course as being prescribed, if no, why they did not complete the course, whether they had used an antibiotic without being prescribed by a doctor or dentist within 12 months prior to the study period (self-medication), frequency of self-medication with an antibiotic during the last year, conditions for which the antibiotics are being self-prescribed, and whether they had ever given someone else an antibiotic that was not prescribed for them. The ninth question included six statements, respondents were asked to tick all that relates to their self-medication with antibiotics. These statements included the following: used antibiotics that were originally prescribed for an infection which recurred later, originally prescribed for another type of infection, obtained from a pharmacy abroad without a prescription, obtained from a pharmacy within Kuwait without a prescription, originally prescribed for another family member, and originally prescribed for someone else who was not a family member. The third section consisted of 13 statements to evaluate respondents’ knowledge about antibiotics in four aspects: action and use (6 statements), side effects (3 statements), and resistance (4 statements). A five-point Likert scale (1 = strongly disagree; 5 = strongly agree) was used to evaluate the participants’ responses (Table 2). Seven attitude statements were included in section four, and respondents were required to answer according to a 5-point Likert scale ranging from “strongly disagree” to “strongly agree’ (Table 3). Finally, the fifth section included 6 statements to explore the doctors’ habits and the patient-doctor relationship regarding prescribing of antibiotics, and responses were measured using a 5-point Likert scale ranging from “strongly disagree” to “strongly agree’ (Table 4).

**Table 1:** General Characteristics of Respondents (n = 680).

Characteristic	Frequency	Percentage (%)
Gender		
Male	213	31.3
Female	467	68.7
**Age (years)**		
21–29	305	44.9
30–39	163	23.9
40–49	113	16.6
50–59	66	9.7
≥ 60	33	4.9
**Marital status**		
Single^⊥^	284	41.8
Married	396	58.2
**Education level**		
Low—intermediate education	85	12.5
High education	595	87.5
**Employment**		
Unemployed*	272	40.0
Employed	408	60.0
**Residence**		
Hawalli	264	38.8
Capital	197	29.0
Mubarak Al-Kabeer	68	11.8
Al-Farwaniyah	47	10.0
Al-Ahmadi	24	6.9
Al-Jahra	80	3.5
**Monthly income**		
< 500 Kuwaiti Dinars (KD)	149	21.9
500–1000 Kuwaiti Dinars (KD)	216	31.8
> 1000 Kuwaiti Dinars (KD)	315	46.3
**Personal health**		
Excellent	267	39.3
Very good	282	41.4
Good	131	19.3
**Work or study in the medical field**		
Yes^	126	18.5
No	554	81.5

^⊥^: Includes divorced and widowed;

*: Includes retired, housewives and students;

^: Includes healthcare professionals and students.

**Table 2:** Respondents’ Knowledge regarding Antibiotics (n = 680).

	Strongly disagree n (%)	Disagree n (%)	Neutral (Uncertain) n (%)	Agree n (%)	Strongly agree n (%)	Median* (IQR)
**A. Action and use**						
**Correct statements**						
1. Different antibiotics are needed to cure different diseases.	13 (1.9)	29 (4.3)	132 (19.4)	373 (54.9)	133 (19.6)	4.0 (1.0)
2. Antibiotics are effective against bacteria	14 (2.1)	42 (6.2)	172 (25.3)	304 (44.7)	148 (21.8)	4.0 (1.0)
3. Antibiotics can kill the bacteria that normally live on the skin and in the gut	22 (3.2)	46 (6.8)	265 (39.0)	265 (39.0)	82 (12.1)	4.0 (1.0)
**Incorrect statements**						
4. Antibiotics speed up the recovery from most coughs and colds	47 (6.9)	102 (15.0)	161 (23.7)	295 (43.4)	75 (11.0)	4.0 (1.0)
5. Antibiotics work on most coughs and colds	44 (6.5)	127 (18.7)	142 (20.9)	303 (44.6)	64 (9.4)	4.0 (2.0)
6. Antibiotics are effective against viruses	122 (17.9)	81 (11.9)	163 (24.0)	246 (36.2)	68 (10.0)	3.0 (2.0)
**B. Side effects**						
7. If you get side effects during a course of antibiotics treatment you should stop taking them as soon as possible	11 (1.6)	21 (3.1)	81 (11.9)	369 (54.3)	198 (29.1)	4.0 (1.0)
8. If you get some kind of skin reaction when using an antibiotic, you should not use the same antibiotic again	29 (4.3)	29 (4.3)	93 (13.7)	327 (48.1)	202 (29.7)	4.0 (1.0)
9. Antibiotics can cause imbalance in the body’s own bacterial flora	7 (1.0)	28 (4.1)	244 (35.9)	296 (43.5)	105 (15.4)	4.0 (1.0)
**C. Resistance**						
**Correct statements**						
10. The unnecessarily use of antibiotics can increase the resistance of bacteria to them	15 (2.2)	77 (11.3)	255 (37.5)	249 (36.6)	84 (12.4)	3.0 (1.0)
11. Resistance to antibiotics is a worldwide problem	15 (2.2)	49 (7.2)	293 (43.1)	222 (32.6)	101 (14.9)	3.0 (1.0)
12. The use of antibiotics among animals can reduce the effect of antibiotics among humans	31 (4.6)	55 (8.1)	443 (65.1)	130 (19.1)	21 (3.1)	3.0 (0)
**Incorrect statement**						
13. Humans can be resistant to antibiotics	16 (2.4)	35 (5.1)	246 (36.2)	309 (45.4)	74 (10.9)	4.0 (1.0)

n: Number of participants;

*1: Strongly disagree; 2: Disagree; 3: Neutral (uncertain); 4: Agree; 5: Strongly agree; IQR: Interquartile range.

**Table 3:** Respondents’ Attitudes towards Use of Antibiotics (n = 680).

	Strongly disagree n (%)	Disagree n (%)	Neutral (Uncertain) n (%)	Agree n (%)	Strongly agree n (%)	Median* (IQR)
**Positive attitude item**						
1. I always complete the course of treatment with antibiotics even if I feel better	46 (6.8)	117 (17.2)	126 (18.5)	246 (36.3)	145 (21.3)	4.0 (1.0)
**Negative attitude items**						
2. It is good to be able to get antibiotics from relatives or friends without having to see a medical doctor.	279 (41.0)	242 (35.6)	82 (12.1)	63 (9.3)	14 (2.1)	2.0 (1.0)
3. I prefer to be able to buy antibiotics from the pharmacy without a prescription.	236 (34.7)	217 (31.9)	116 (17.1)	93 (13.7)	18 (2.6)	2.0 (2.0)
4. I prefer to keep antibiotics at home in case there may be a need for them later	162 (23.8)	217 (31.9)	97 (14.3)	164 (24.1)	40 (5.9)	2.0 (2.0)
5. If I feel better after a few days, I sometimes stop taking my antibiotics before completing the course of treatment	174 (25.6)	200 (29.4)	83 (12.2)	183 (26.9)	40 (5.9)	2.0 (3.0)
6. I prefer to use an antibiotic if I have a cough for more than a week	96 (14.1)	196 (28.8)	150 (22.1)	203 (29.9)	35 (5.1)	3.0 (2.0)
7. When I have a sore throat I prefer to use an antibiotic	49 (7.2)	132 (19.4)	162 (23.8)	281 (41.3)	56 (8.2)	3.0 (2.0)

n: Number of participants;

*1: Strongly disagree; 2: Disagree; 3: Neutral (uncertain); 4: Agree; 5: Strongly agree; IQR: Interquartile range.

**Table 4:** Doctor’s habit and the patient/doctor relationship (n = 680).

	Strongly disagree n (%)	Disagree n (%)	Neutral (Uncertain) n (%)	Agree n (%)	Strongly agree n (%)	Median* (IQR)
1. Pharmacists often tell you how antibiotics should be used	40 (5.9)	90 (13.2)	115 (16.9)	339 (49.9)	95 (14.0)	4.0 (1.0)
2. Doctors often take time to inform the patient during the consultation how antibiotics should be used	113 (16.6)	230 (33.8)	205 (30.1)	114 (16.8)	18 (2.6)	2.0 (1.0)
3. I trust the doctor decision if she or he decides not to prescribe antibiotic	30 (4.4)	62 (9.1)	148 (21.8)	337 (49.6)	103 (15.1)	4.0 (1.0)
4. I trust the doctor’s decision when she or he prescribes antibiotics	39 (5.7)	95 (14.0)	190 (27.9)	302 (44.4)	54 (7.9)	4.0 (1.0)
5. Doctors often prescribe antibiotics because the patient expects it	41 (6.0)	120 (17.6)	161 (23.7)	288 (42.4)	70 (10.3)	4.0 (1.0)
6. Doctors often take time to consider carefully whether antibiotics are needed or not	113 (16.6)	185 (27.2)	142 (20.9)	201 (29.6)	39 (5.7)	3.0 (2.0)

n: Number of participants;

*1: Strongly disagree; 2: Disagree; 3: Neutral (uncertain); 4: Agree; 5: Strongly agree; IQR: Interquartile range.

### Statistical analysis

Data were entered into the Statistical Package for Social Sciences (SPSS, version 21, SPSS, Chicago, IL, U.S.A.) and descriptive analysis conducted. The results were reported as percentage (95% confidence interval) and median (Interquartile range). To simplify the results’ presentation in the text, those who answered “strongly agree” or “agree” were classified as “agreed”, and those who answered “strongly disagree” or “disagree” as having disagreed. The internal consistency for the sections to determine knowledge of and attitude towards use of antibiotics was assessed using Cronbach’s α test. The test results were as follows: 13 statements of knowledge about antibiotics, 0.72; and 7 statements of attitude towards use of antibiotics, 0.71. A scoring system was applied to measure the respondents’ knowledge and attitudes towards antibiotics. (i) The antibiotic knowledge score was calculated as a continuous variable by summing the participant’s number of correct responses to 13 statements. One point was awarded for each correct response (strongly agree or agree for correct statement and strongly disagree or disagree for incorrect statement), and zero for each wrong or uncertain response, with a maximum obtainable correct score of 13 for each respondent. The knowledge score was categorized into three levels indicated by low (0–6), moderate (7–10), and high (11–13). (ii) The attitude score was calculated as a continuous variable by summing the participant’s number of appropriate responses to 7 statements. One point was awarded for each appropriate response (strongly agree or agree for positive statement and strongly disagree or disagree for negative statement) and zero for each inappropriate or uncertain response, with a maximum obtainable correct score of 7 for each respondent. The attitude score was categorized into two levels indicated by negative (0–3) and positive (4–7).

Association of respondents’ characteristics with antibiotic prescribing, self-medication with an antibiotic, knowledge of antibiotics and attitudes towards antibiotic use was first evaluated using univariate logistic regression. All variables with p < 0.25 in the univariate analysis were included in the multivariate logistic regression analysis to determine the factors that are independently associated with each of the four dependent variables. Only the results of multivariate logistic analysis are reported showing odds ratio (OR) and 95% confidence interval. Statistical significance was accepted at p < 0.05. The dependent variables: (1) antibiotic being prescribed in the last year (0 = no, 1 = yes); (2) self-medicated with an antibiotic in the past year (0 = no, 1 = yes); (3) knowledge of antibiotics (0 = incorrect response [score 0–6], 1 = correct response [score 7–13]); and (4) attitudes towards antibiotics use (0 = negative attitude [score 0–3], 1 = positive attitude [score 4–7]). In all models the following independent variables were included: (1) gender: males and females; (2) age: [21–29 years], [30–39 years], [40–49 years], [50–59 years], ≥ 60 years; (3) marital status: single and married; (4) level of education: low-intermediate [0–12 years] for those who completed secondary school or less, and high [>12 years] for those who had diploma, or bachelor degree or postgraduate degree; (5) employment: unemployed and employed; (6) residence: (7) monthly income: low [< 500 Kuwaiti Dinars (KD)], middle [500–1000 KD] and high [> 1000 KD]; (8) work or study in a health-care related [yes, no]; and (9) self-rated health status: excellent, very good, good. Spearman rank correlation was also used to analyze the association between the four dependent variables. Due to large number of statistical associations that were tested in this study there was a possibility of spurious significances. Hence, Benjamini-Hochberg-Yekutieli (FDR) adjusted p values were computed using R software and reported along with the unadjusted p values. An FDR score < 0.05 was considered as statistically significant.

## Results

A total of 770 Kuwaiti subjects were approached to be included in the study, 680 agreed to participate giving a response rate of 88.3%. Their median (interquartile range, IQR) age was 30.5 (19) years. Of the 680 respondents, 68.7% were females and 87.5% had high education. Table 1 summarizes the general characteristics of respondents. Seven-in-ten responders (n = 495; 72.8%; 95% CI: 69.3–76.1) stated that they had been prescribed an antibiotic within 12 months preceding the survey. More than two-fifths (n = 226; 45.7%; CI: 41.2–50.2) of them reported the use of prescribed antibiotics two to five times in the past year, followed by 31.7% once, 4.6% six to ten times, and 3.6% more than 10 times. Seventy one (14.3%) did not remember the frequency of being prescribed antibiotics within the last year. On the multivariate logistic regression analysis, the findings revealed that one independent variable was significantly associated with antibiotic prescribing. Respondents with excellent self-rated health status were less likely to be prescribed an antibiotic (OR: 0.42; CI: 0.24–0.74; p = 0.002; FDR score = 0.022) compared to those with either very good or good self-rated health status. When asked if they did take their last antibiotic course as being prescribed, about two-thirds (n = 321; 64.9%; CI: 60.4–69.0) completed the antibiotic course; however, 174 (36.0%; CI: 31.8–40.4) had not finished the course. The main reason was that they felt better (n = 118; 67.8%; CI: 60.3–74.6), followed by forgot to take the antibiotic (n = 42; 24.1%; CI: 18.3–31.3), and side effects that made them feel unwell (n = 14; 8.1%; CI: 4.6–13.4).

One hundred and eighty seven (27.5%; CI: 24.2–31.1) of the study population had used antibiotics without a medical consultation within 12 months prior to the study period. Most of them (n = 166; 88.8%; CI: 83.1–92.8) were also prescribed an antibiotic within 12 months prior to the study period. The majority of them (n = 118; 63.1%; CI: 55.7–69.9) were self-medicated with the antibiotics two to five times within 12 months prior to the study period. On the multivariate logistic regression analysis, there were no significant associations between the respondents’ characteristics and self-medication with antibiotics. Self-medication was found to be significantly correlated with being prescribed an antibiotic within 12 months prior to the study period (p < 0.001; FDR score = 0.006) and attitudes towards antibiotics’ use (p < 0.001; FDR score = 0.006). Respondents more likely to be self-medicated with antibiotics were those who reported being prescribed an antibiotic within 12 months prior to the study period and those who had negative attitude towards use of antibiotics. There was no significant relationship between self-medication and respondents’ knowledge of antibiotics. Ninety seven (51.9%) of the self-medicated responders indicated that they had given an antibiotic to someone else to use without a medical consultation. One hundred and nineteen (63.6%) stated that they used antibiotics that were originally prescribed for an infection which recurred later, and 21 (11.2%) for another type of infection. Fifty nine (31.6%) of the respondents who had self-medicated obtained the antibiotics directly from private pharmacies in Kuwait, and from pharmacies abroad 20 (10.7%) without a prescription. Other sources of obtaining antibiotics included family members (n = 50, 26.7%) and friends (n = 7; 3.7%). The self-medication with antibiotics was mainly to treat common cold (n = 102; 54.5%), sore throat (n = 77; 41.2%), cough (n = 46; 24.6%), genitourinary infections (n = 13; 7%), and superficial wounds (n = 12; 6.4%).

The median (IQR) knowledge score of respondents was 7 (4.0) out of a maximum score of 13 [moderate knowledge]: 2.8% (n = 19) did not indicate any correct response, whereas 43.8% (n = 298) listed one to six correct responses, 43.4% (n = 295) seven to ten, and 10% (n = 68) eleven to thirteen correct responses. Table 2 presents the respondents’ knowledge regarding antibiotics. The majority of respondents correctly agreed on two statements related to safety and one statement about the use of antibiotics: ‘if you get side effects during a course of antibiotics treatment you should stop taking them as soon as possible’ (83.4%; CI:80.3–86.1), ‘if you get some kind of skin reaction when using an antibiotic, you should not use the same antibiotic again’ (77.8%; CI:74.4–80.83), and ‘different antibiotics are needed to cure different diseases’ (74.4%; CI: 70.9–77.6).

In contrast, participants were less knowledgeable about whether antibiotics were effective against coughs and colds, viruses, bacteria and normal flora. Over half of the respondents incorrectly agreed that ‘antibiotics speed up the recovery from most coughs and colds’ (n = 370; 54.4%; CI: 50.6–58.2) and ‘antibiotics work on most coughs and colds’ (54.0%; CI: 50.1–57.8). Confusion about whether antibiotics were effective against bacteria or viruses was existing in this study. One third (33.5%; CI: 30.1–37.2) of participants did not agree that ‘antibiotics are effective against bacteria’ and 46.2% (CI: 42.4–50.0) incorrectly agreed that ‘antibiotics are effective against viruses’. Almost 40% of those agreed that antibiotics are effective against bacteria also agreed that antibiotics are effective against viruses. Over two-fifths of respondents did not agree that antibiotics can cause imbalance in the body’s own bacterial flora (41%; CI: 37.3–44.8) and that antibiotics can kill the bacteria that normally live on the skin and in the gut (49.0%; CI: 45.2–52.8). Further, the study population were also less knowledgeable about antibiotic resistance. Over half of participants did not agree on the statements: ‘the unnecessarily use of antibiotics can increase the resistance of bacteria to them’ (51%; CI: 47.2–54.8), ‘resistance to antibiotics is a worldwide problem’ (52.5%; CI: 48.7–56.3), and ‘the use of antibiotics among animals can reduce the effect of antibiotics among humans’ (77.8%; CI: 74.4–80.8). Almost six-in-ten responders incorrectly agreed that humans can be resistant to antibiotics’ (56.3%; CI: 52.5–60.8).

On the multivariate logistic regression analysis, the findings revealed that respondents who work or study in a health-care related field were found to be more knowledgeable than those who did not work or study in a health-care related field (OR: 11.33; CI: 6.14–20.98; p<0.001; FDR score = 0.011). There were no significant associations between respondents’ knowledge about antibiotics and the other independent variables.

Table 3 presents the respondents’ attitudes towards use of antibiotics. The median (IQR) attitude score of respondents was 4 (3.0) out of a maximum score of 7 [positive attitude]: 7.1% (n = 48) did not indicate any appropriate response, whereas 34.1% (n = 232) indicated one to three appropriate responses, 36.9% (n = 251) four to five, and 21.9% (n = 149) six to seven appropriate responses. Over half of respondents expressed positive attitudes towards not to get antibiotics from relatives or friends without a medical consultation (76.6%; CI: 73.2–79.7), not to obtain antibiotics from the pharmacy without a prescription (66.6%; CI: 62.9–70.1), not to keep antibiotics at home for future use (55.7%; CI: 51.9–59.5), and to always complete the antibiotic course (57.6%; CI: 53.7–61.2). Nevertheless, the higher rates of negative attitudes were related to the use of antibiotics for treatment of sore throat (73.4%; CI: 69.9–76.6) and cough (57.1%; CI: 53.2–60.8).

The multivariate logistic regression analysis revealed that two independent variables have a significant influence on the respondents’ overall attitude towards use of antibiotics. Respondents with high education and those who work or study in a health-care related field were found to express more positive attitude than those with lower education (OR: 2.07; CI:1.23–3.47; p = 0.006; FDR score = 0.042) and those who did not work or study in a health-care related field (OR: 2.05; CI: 1.30–3.23; p = 0.002; FDR score = 0.022).

A significantly higher percentage of participants agreed that pharmacists often tell them how antibiotics should be used than the doctors during the consultation (P <0.001; FDR score = 0.006). A significantly greater percentage of respondents indicated trusting the doctor who is not prescribing an antibiotic (i.e., low antibiotic prescriber), than the doctor prescribing an antibiotic (i.e., high antibiotic prescriber) (p<0.001; FDR score = 0.006). Five-in ten responders (52.7%) agreed that doctors often prescribe an antibiotic because the patient expects it. Only 35.3% (CI: 31.7–39.0) of respondents agreed that doctors often take time to consider carefully whether antibiotics are needed or not.

## Discussion

This is the first known study to be conducted in Kuwait, and probably in the GCC countries to comprehensively demonstrate knowledge, attitudes, and practice towards antibiotic use among the public. The present findings would be the first step in providing a baseline quantitative data of patterns of antibiotics use, knowledge and attitudes regarding antibiotics among Kuwaiti citizens. This will aid in the assessment of the adequacy of the present community educational campaigns on antibiotics, and provide further insight in designing future multifaceted interventions targeting specific areas to promote rational antibiotic use, and replenish the knowledge and attitude gaps as an effort against antibiotic resistance.

High rates of antibiotic prescribing were identified by the present study. These results are close to previous studies from Italy, Syria and Jordan, [20,21,24] but higher than that in the UK, Malaysia and European countries [15,16,22,23,26]. This high rate of prescribing could partly be explained by the respondents agreement that doctors often prescribe an antibiotic because the patient expects it and disagreement that doctors often take time to consider carefully whether antibiotics are needed or not. It is well documented that overprescribing by physicians even in the absence of appropriate indications due to diagnostic doubt, their lack of knowledge regarding optimal therapies, and patient demand are factors contributing to the occurrence and increase of antibiotic resistance [2,7,10,11]. In fact, numerous reports have shown that patient’s expectation is a crucial factor for antibiotic prescribing and that antibiotics are more likely to be prescribed under patient pressure [29–31]. Moreover, it was reported that physicians often prescribe antibiotics because they perceive that patients want them despite their opinion that antibiotics are not required [29]. Nevertheless, unnecessary prescribing is also related to the imprecise and overestimation of patients’ expectations as physicians do not usually discuss patients’ demands for antibiotics directly, and the demand is often assumed [30]. This underscores the need for policies of auditing antibiotic prescriptions in the health care facilities of Kuwait, and investigating the consultation behavior and other behavioral components engaged in patients’ expectations for antibiotics. Furthermore, the present finding that about two-thirds of participants stated their trust in doctors who are not prescribing antibiotics should be utilized in designing effective interventions to reduce patients’ expectations from antibiotics and to increase knowledge about antibiotic resistance. It was reported that patients were satisfied with improved understanding of their diseases even if an antibiotic was not prescribed [30,32].

More than one-third of those being prescribed an antibiotic did not complete their last antibiotic course as prescribed, most of them stopped treatment because they felt better. In addition, 45% of respondents expressed negative attitude to stop taking antibiotics before completing the course once they felt better. This misconception in the antibiotic use may put the patient at risk of relapse with resistant pathogenic bacteria. It is widely believed that inadequate dosing, incomplete courses, and indiscriminate drug use have contributed to the emergence and spread of antibiotics resistance, which is a current problem in Kuwait [2,9–11].

The current finding that almost three-in-ten of the study population had self-medicated with antibiotics is close to that reported from Italy and 11 European countries [20,33]. However, it is worthy to mention that it is lower than that reported in studies from the Middle Eastern countries namely, Jordan, United Arab Emirates, Palestine, Lebanon, Iraq and Yemen, which ranged between 40.7% and 78% [24,34–39]. In contrast, it is higher than the rates in Hong Kong, the UK, Malaysia, European countries and Indonesia, which ranged between 4.8% and 9% [5,15,22,23,26,40]. The lower rate of self-medication in Kuwait compared to other countries in the region could be partly explained by the fact that Kuwait has a policy that prohibits the dispensing of antibiotic without a prescription. In the present study, 31.6% of the self-medicated responders obtained the antibiotics directly from private pharmacies in Kuwait without prescriptions, which is considerably lower than that reported from other countries in the Middle East region that ranged between 53.6% and 96.7% [24,34–39]. Furthermore, one-third of the study population expressed negative attitude to buy antibiotics from the pharmacy without a prescription. Hence, the current study highlights the need for further enforcement of regulations. It was reported that patients’ demands and the profit interest of the private pharmacies are factors that may lead to inappropriate dispensing of antibiotics without a prescription. Hence, a balance between professionalism and profitable commercial aims needs to be addressed [22]. Moreover, the present finding that more of the respondents declared that they obtained information about antibiotic use from pharmacists than from physicians highlights that pharmacists can have a vital role to play in public education about the prudent use of antibiotics. Implementation of pharmaceutical care in community pharmacies can help to improve public knowledge and attitude towards antibiotics, which is at present, poorly developed in Kuwait. Community pharmacists are the most accessible health care providers to the public, and can be utilized in contributing to public knowledge about proper antibiotic use.

The present findings reveal that other sources of obtaining antibiotics included family members and friends, and over half of the self-medicated responders indicated that they had given an antibiotic to someone else to use without a medical consultation. Moreover, 44.3% of the study participants expressed negative attitude to keep left-over antibiotics for future use, and 23.3% to get antibiotics from relatives or friends without advice from a physician. These findings demonstrated that a proportion of Kuwaiti individuals share used antibiotics with others, thus subjecting the general population to the problem of antibiotics misuse.

Respondents self-medicated with antibiotics for minor ailments such as common cold, sore throat, and cough, all of which can be self-limiting with the appropriate medical and supportive care. In addition, over 60% of participants expressed negative attitude to take antibiotics for treatment of sore throat or cough. This could be explained by the low respondents’ knowledge about whether antibiotics were effective against coughs and colds, viruses, and bacteria. Over half of them incorrectly thought that antibiotics work on most coughs and colds and speed up their recovery, which is comparable to the rates reported in Jordan and South Korea, [24,25] but considerably higher than reports from the UK, Sweden, Italy, and the USA [15–17,20,41]. Furthermore, the present study revealed confusion among respondents about whether antibiotics are effective against bacteria or viruses. Seventy percent of participants failed to identify that antibiotics have no significant therapeutic effects on viruses, which is consistent with the rates in Malaysia [22,23]. In contrast, lower proportions were reported in other studies from the UK, Sweden, Jordan, and European countries [15,17,24,26]. Two-thirds of the study population agreed that antibiotics are effective against bacteria, which is higher than that reported in the UK and Jordan, [15,24] but lower than previous rates in Sweden and Malaysia [17,22]. These findings demonstrate clear misconceptions and confusions among the study population regarding the role of antibiotics, and about the cause of the disease whether it is a viral or bacterial illness. This study pointed out that the recognition of antibiotic resistance as a global problem and the factors responsible for it remain largely unknown among the public in Kuwait. The prevalence of the poor participants’ knowledge about resistance is considerably higher than the rates in Hong Kong, the UK, Sweden, and Malaysia [5,15,17,22]. Moreover, the prevalence of negative attitude in the present study is higher compared to previous studies from Hong Kong the UK, Sweden, the USA, and Malaysia [5,15–18,22,23]. The current findings highlight the need for public education about effectiveness and resistance of antibiotics. Also it is important that physicians use the terms “bacteria” and “virus” during the explanation of the prescribing decision [17]. The public educational campaigns should not only provide information, but also deliver proper and practical ways to alter their attitudes and behavior to rationalize antibiotic use. It has been reported that increasing community knowledge without imparting the appropriate attitude can result in higher incidences of antibiotic misuse [16].

In the present study, there were no significant associations between self-medication with antibiotics and the background characteristics of the respondents. In contrast, previous studies reported significant association between self-medication and age, gender, level of education and income [20,24,33–39]. The current finding that those who were prescribed antibiotics within 12 months prior to the study period were more likely to self-medicate suggest that physicians should be involved to strengthen the public education campaigns since it has been shown that effective doctor-patient communication and patient empowerment reduced inappropriate antibiotic use [20]. The finding that self-medication is prevalent among those with negative attitudes towards antibiotic use, and is not associated with knowledge is contrary to a previous report from the UK, where people with a better knowledge of and attitude to antibiotics were more likely to be self-medicated [15]. The non-significant association between self-medication and antibiotic knowledge suggests that education only campaigns that emphasize knowledge may not be effective to decrease self-medication among Kuwaitis. This finding has interesting implications for the development of comprehensive and multifaceted interventions including: (i) focused education to reduce the public misconceptions about antibiotics use for minor ailments and to change their attitude to limit self-medication, and (ii) stricter governmental regulation directed at diminishing the antibiotic availability without a prescription, and at enabling the dispensation of the exact numbers of antibiotic tablets or capsules.

Respondents’ adequate knowledge of antibiotics was identified to correlate positively with attitude, which is consistent with previous studies, where appropriate knowledge of antibiotics was identified to be a predictor for positive attitude towards antibiotic use [23,25]. The present findings identified groups of respondents who were prone to self-medication, low knowledge and negative attitude towards antibiotics. Hence, efforts should be focused to reach these groups of individuals in future public education campaigns to improve knowledge and change attitudes and behaviors towards antibiotic use. 2010 Eurobarometer findings demonstrate that targeted media campaigns at those with low knowledge are more likely to change their usage habits. Also, the involvement of physicians and pharmacists to have a key role in changing public views and behavior would strengthen the education campaigns [26].

Interpretation of the findings of this study should take into account certain potential limitations that might impact upon its conclusions. While efforts were made to obtain representative samples, the over representation of female gender and higher educational level in the study sample indicates selection bias. That would have affected the external validity in terms of generalizing the findings to the wider population. We acknowledge that this type of study, using a self-administered questionnaire, has its limitations. It depends very much upon information given by respondents and open to recall bias. It is possible that respondents may over-report socially desirable behaviors or under-report socially undesirable behaviors. The extent of truthful answers or verifying respondents’ claims is not possible in this type of study, which were taken at face value. The completion of the questionnaires anonymously and assurance of confidentiality would minimize the over- and under-reporting. A further limitation is the cross-sectional nature of the data that represented one point in time and, therefore, do not reflect any changes in respondents’ knowledge and attitude over time in relation to antibiotics use. Despite these limitations, the present findings provide important information for evaluating and improving knowledge, attitude and practice towards antibiotics use.

## Conclusions

The present findings allow for important comparative work with existing and future investigations in middle-eastern countries, and worldwide. Given the rapid growth rate of antibiotic resistance in Kuwait, and documented health issues related to inappropriate use of antibiotics, the present findings highlight important concerns regarding antibiotics among Kuwaiti citizens. The key findings of this study will help policy makers in Kuwait to plan and establish future effective multifaceted interventions to improve the appropriate use of antibiotics. These should include the followings: (i) policies of auditing antibiotic prescriptions in the healthcare facilities, and investigating the consultation behavior and other behavioral components engaged in patients’ expectations for antibiotics, (ii) improve communication about antibiotic appropriateness between healthcare professionals and patients, (iii) public education programs using all media means that should be targeted more efficiently at those who have low knowledge, negative attitude and inappropriate practice towards antibiotic use. These campaigns should not only disseminate information, but also use behavioral and persuasion theories and repetition to target modifications in health risk attitudes. Every campaign should be followed by an evaluation of its effectiveness, (iv) highlighting pharmacists’ role in health education and promotion, and responsibility in prohibiting antibiotic dispensing without prescription, and (v) monitoring sources of obtaining antibiotics through enforcing the strict regulations. Further research to investigate knowledge, attitude and practice towards antibiotic use among expatriates will help in the adoption and implementation of successful future policies to promote rational antibiotic use among the public in Kuwait. (S1 Dataset)

## Supporting Information

S1 Dataset KAP towards Antibiotic use among the Public in Kuwait. (SAV)

## References

[1] World Health Organization (2007) The world health report 2007. A safer future: global public health security in the 21st century. Available: http://www.who.int/whr/2007. Accessed 11 July 2014.

[2] World Health Organization (2001) WHO Global Strategy for Containment of Antimicrobial Resistance. WHO/CDS/CSR/DRS/2001.2. Available: http://www.who.int/csr/resources/publications/drugresist/en/EGlobal_Strat.pdf. Accessed 11 July 2014.

[3] Costelloe C, Metcalfe C, Lovering A, Mant D, Hay AD (2010) Effect of antibiotic prescribing in primary care on antimicrobial resistance in individual patients: systematic review and meta-analysis. BMJ 340: c2096 10.1136/bmj.c2096 20483949

[4] Vaananen MH, Pietila K, Airaksinen M (2006) Self-medication with antibiotics—does it really happen in Europe? Health policy 77: 166–171. 16095749 10.1016/j.healthpol.2005.07.001

[5] You JH, Yau B, Choi KC, Chau CT, Huang QR, et al (2008) Public knowledge, attitudes and behavior on antibiotic use: a telephone survey in Hong Kong. Infection 36: 153–157. 10.1007/s15010-007-7214-5 18231717

[6] Davey P, Pagliari C, Hayes A (2002) The patient’s role in the spread and control of bacterial resistance to antibiotics. Clin Microbiol Infect 8: 43–68. 12427207 10.1046/j.1469-0691.8.s.2.6.x

[7] Franco BE, Altagracia Martinez M, Sanchez Rodriguez MA, Wertheimer AI (2009) The determinants of the antibiotic resistance process. Infect Drug Resist 2: 1–11. 21694883 PMC3108730

[8] Hulscher ME, van der Meer JW, Grol RP (2010) Antibiotic use: how to improve it? Int J Med Microbiol 300: 351–356. 10.1016/j.ijmm.2010.04.003 20434950

[9] Zhang R, Eggleston K, Rotimi V, Zeckhauser RJ (2006) Antibiotic resistance as a global threat: evidence from China, Kuwait and the United States. Global Health 2: 6 16603071 10.1186/1744-8603-2-6 PMC1502134

[10] Memish ZA, Ahmed QA, Arabi YM, Shibl AM, Niederman MS (2007) Microbiology of community-acquired pneumonia in the Gulf Corporation Council states. J Chemother 19: 17–23. 18073166 10.1080/1120009x.2007.11782430

[11] Aly M, Balkhy HH (2012) The prevalence of antimicrobial resistance in clinical isolates from Gulf Corporation Council countries. Antimicrob Resist Infect Control 1: 26 10.1186/2047-2994-1-26 22958584 PMC3436690

[12] Awad A, Al-Saffar N (2010) Evaluation of drug use practices at primary healthcare centers of Kuwait. Eur J Clin Pharmacol 66: 1247–1255. 10.1007/s00228-010-0872-8 20669012

[13] Al-Mousa HH, Aly NY (2012) Kuwait national campaign for proper use of antibiotics. Med Princ Pract 21: 97 10.1159/000331903 22025092

[14] Hawkings NJ, Butler CC, Wood F (2008) Antibiotics in the community: a typology of user behaviours. Patient Educ Couns 73: 146–152. 10.1016/j.pec.2008.05.025 18640805

[15] McNulty CA, Boyle P, Nichols T, Clappison P, Davey P (2007) Don’t wear me out—the public’s knowledge of and attitudes to antibiotic use. J Antimicrob Chemother 59: 727–738. 17307770 10.1093/jac/dkl558

[16] McNulty CA, Boyle P, Nichols T, Clappison P, Davey P (2007) The public’s attitudes to and compliance with antibiotics. J Antimicrob Chemother 60: 63–68. 10.1093/jac/dkm161 17656386

[17] Andre M, Vernby A, Berg J, Lundborg CS (2010) A survey of public knowledge and awareness related to antibiotic use and resistance in Sweden. J Antimicrob Chemother 65: 1292–1296. 10.1093/jac/dkq104 20360063

[18] Vanden Eng J, Marcus R, Hadler JL, Imhoff B, Vugia DJ, et al (2003) Consumer attitudes and use of antibiotics. Emerg Infect Dis 9: 1128–1135. 14519251 10.3201/eid0909.020591 PMC3016767

[19] Chan YH, Fan MM, Fok CM, Lok ZL, Ni M, et al (2012) Antibiotics nonadherence and knowledge in a community with the world’s leading prevalence of antibiotics resistance: implications for public health intervention. Am J Infect Control 40: 113–117. 10.1016/j.ajic.2011.03.017 21741119 PMC7115258

[20] Napolitano F, Izzo MT, Di Giuseppe G, Angelillo IF (2013) Public knowledge, attitudes, and experience regarding the use of antibiotics in Italy. PLoS One 8: e84177 10.1371/journal.pone.0084177 24376793 PMC3871686

[21] Barah F, Goncalves V (2010) Antibiotic use and knowledge in the community in Kalamoon, Syrian Arab Republic: a cross-sectional study. East Mediterr Health J 16: 516–521. 20799551

[22] Ling Oh A, Hassali MA, Al-Haddad MS, Syed Sulaiman SA, Shafie AA, et al (2011) Public knowledge and attitudes towards antibiotic usage: a cross-sectional study among the general public in the state of Penang, Malaysia. J Infect Dev Ctries 5: 338–347. 21628809 10.3855/jidc.1502

[23] Lim KK, Teh CC (2012) A Cross Sectional Study of Public Knowledge and Attitude towards Antibiotics in Putrajaya, Malaysia. South Med Rev 5: 26–33. 23532680 PMC3606936

[24] Shehadeh M, Suaifan G, Darwish RM, Wazaify M, Zaru L, et al (2012) Knowledge, attitudes and behavior regarding antibiotics use and misuse among adults in the community of Jordan. A pilot study. Saudi Pharm J 20: 125–133. 10.1016/j.jsps.2011.11.005 23960783 PMC3744980

[25] Kim SS MS, Kim EJ (2011) Public knowledge and attitudes regarding antibiotic use in South Korea. J Korean Acad Nurs 41: 742–749. 10.4040/jkan.2011.41.6.742 22310858

[26] European Commission (2013) Special Eurobarometer 407: Antimicrobial Resistance. Brussels, Belgium: European Commission. Available: http://ec.europa.eu/health/antimicrobial_resistance/docs/ebs_407_en.pdf. Accessed 12 December 2014.

[27] Kuwait Central Statistical Bureau (2011) General Population Census. Available: http://www.csb.gov.kw/Socan_Statistic.aspx?ID=6. Accessed 12 December 2013.

[28] Raosoft Inc (2004) Raosoft Sample Size Calculator. Available: http://www.raosoft.com/samplesize.html. Accessed 12 December 2013.

[29] Butler CC, Rollnick S, Pill R, Maggs-Rapport F, Stott N (1998) Understanding the culture of prescribing: qualitative study of general practitioners’ and patients’ perceptions of antibiotics for sore throats. BMJ 317: 637–642. 9727992 10.1136/bmj.317.7159.637 PMC28658

[30] Ong S, Nakase J, Moran GJ, Karras DJ, Kuehnert MJ, et al (2007) Antibiotic use for emergency department patients with upper respiratory infections: prescribing practices, patient expectations, and patient satisfaction. Ann Emerg Med 50: 213–220. 17467120 10.1016/j.annemergmed.2007.03.026

[31] Kumar S, Little P, Britten N (2003) Why do general practitioners prescribe antibiotics for sore throat? Grounded theory interview study. BMJ 326: 138 12531847 10.1136/bmj.326.7381.138 PMC140007

[32] Hamm RM, Hicks RJ, Bemben DA (1996) Antibiotics and respiratory infections: are patients more satisfied when expectations are met? J Fam Pract 43: 56–62. 8691181

[33] Grigoryan L, Burgerhof JG, Degener JE, Deschepper R, Lundborg CS, et al (2008) Determinants of self-medication with antibiotics in Europe: the impact of beliefs, country wealth and the healthcare system. J Antimicrob Chemother 61: 1172–1179. 10.1093/jac/dkn054 18296694

[34] Abasaeed A, Vlcek J, Abuelkhair M, Kubena A (2009) Self-medication with antibiotics by the community of Abu Dhabi Emirate, United Arab Emirates. J Infect Dev Ctries 3: 491–497. 19762966 10.3855/jidc.466

[35] Al-Ramahi R (2013) Patterns and attitudes of self-medication practices and possible role of community pharmacists in Palestine. Int J Clin Pharmacol Ther 51: 562–567. 10.5414/CP201814 23587151

[36] Cheaito L, Azizi S, Saleh N, Salameh P (2014) Assessment of self-medication in population buying antibiotics in pharmacies: a pilot study from Beirut and its suburbs. Int J Public Health 59: 319–327. 10.1007/s00038-013-0493-y 23942698

[37] Jassim AM (2010) In-home Drug Storage and Self-medication with Antimicrobial Drugs in Basrah, Iraq. Oman Med J 25: 79–87. 10.5001/omj.2010.24 22125705 PMC3215488

[38] Mohanna M (2010) Self-medication with Antibiotic in Children in Sana’a City, Yemen. Oman Med J 25: 41–43. 10.5001/omj.2010.10 22125697 PMC3215380

[39] Sawair FA, Baqain ZH, Abu Karaky A, Abu Eid R (2009) Assessment of self-medication of antibiotics in a Jordanian population. Med Princ Pract 18: 21–25. 10.1159/000163041 19060486

[40] Widayati A, Suryawati S, de Crespigny C, Hiller JE (2011) Self medication with antibiotics in Yogyakarta City Indonesia: a cross sectional population-based survey. BMC Res Notes 4: 491 10.1186/1756-0500-4-491 22078122 PMC3273454

[41] Belongia EA, Naimi TS, Gale CM, Besser RE (2002) Antibiotic use and upper respiratory infections: a survey of knowledge, attitudes, and experience in Wisconsin and Minnesota. Prev Med 34: 346–352. 11902851 10.1006/pmed.2001.0992

